# Oleuropein impact on colorectal cancer

**DOI:** 10.2144/fsoa-2023-0131

**Published:** 2024-05-23

**Authors:** Hamdi Nsairat, Areej M Jaber, Haya Faddah, Somaya Ahmad

**Affiliations:** 1 Pharmacological & Diagnostic Research Center, Faculty of Pharmacy, Al-Ahliyya Amman University, Amman, 19328, Jordan

**Keywords:** apoptosis, colorectal, *Olea europaea*, oleuropein, olive oil

## Abstract

Colorectal cancer (CRC) is considered the third most common cancer in the world. In Mediterranean region, olives and olive oil play a substantial role in diet and medical traditional behaviors. They totally believe that high consumption of olive products can treat a wide range of diseases and decrease risk of illness. Oleuropein is the main active antioxidant molecule found in pre-mature olive fruit and leaves. Recently, it has been demonstrated that oleuropein is used in cancer therapy as an anti-proliferative and apoptotic agent for some cancer cells. In this review, we would like to explore the conclusive effects of oleuropein on CRC with respect to *in vitro* and *in vivo* studies.

## Abstract (plain language summary)

Colorectal cancer (CRC) is considered the third most common cancer in the world.

Colorectal cancer treatment options involve targeted therapy, immune- and gene therapy.

Consumption of olive products can treat a wide range of diseases and decrease risk of illness.

Oleuropein is the primary effective antioxidant molecule in pre-mature olive fruits and leaves.

Oleuropein was proven to be used in cancer treatment as anti-proliferative and an apoptotic enhancer in several cancer cells.

Several biological receptors were verified to be involved in OLR action against CRC.

OLR suppress the proliferation of CRC cells by preventing DNA generation and protein expression.

OLR blocked the growth and diversity of 84% of colonic tumors.

OLR has a good synergistic effect when merged with anticancer agents.

High concentrations of OLR administered *in vivo* were demonstrated to be safe, and studies on rodents showed that it had an excellent safety profile.

The third most prevalent cancer in the world by incidence is colorectal cancer (CRC) [1]. The development of colon cancer is influenced by genetic mutation, lifestyle or environmental factors or unhealthy dietary habits [2]. Colon cancer begins usually in the large intestine due to the uncontrolled proliferation of colon or rectum cells and start to spread to the lower portion of the digestive tract. Chemotherapy, laparoscopic surgery, immunotherapy, radiotherapy and targeted therapy are among the treatment options mostly used to treat primary and metastatic colon cancer [^3-6^].

Chemotherapy is considered the core treatment option for metastases in the CRC patients with increased survival rate. Chemotherapy has numerous problems, involving systemic toxicity, an inadequate response time, unpredictability in acquired and innate resistance and a lack of tumor selectivity [7]. The lack of the ability to improve patient outcomes in managing colorectal metastases, and raised mortality rates encourage researchers to search for alternative treatment approaches such as medicinal plants or via drug delivery systems [^8-10^].

Plant-derived phenolic molecules are the most significant active compounds involved in anti-colorectal cancer actions [11,12]. Oleuropein (OLR) is the most efficient phenolic molecule for reducing cancer cell viability in various categories of cancer as breast, gastric and kidney [13,14]. OLR is a phenolic substance that is mostly discovered in various parts of the olive tree, mainly in their leaves [15,16]. OLR manifests anti-inflammatory, anti-cancer and antioxidant properties. OLR was reported to decrease cyclooxygenase-2 and IL-17 expression and attenuates inflammatory damage in colonic samples from ulcerative colitis patients [17]. OLR also demonstrated a controversial modulatory action on inflammation *in vitro* when tested at concentrations exceeding those detectable in human plasma. OLR had no effect on lipopoly saccharides-triggered release of TNF-α, IL-6 and IL-8, but 5 μM HT reduced IL-10 secretion [18].

Based on several studies related to CRC, OLR clearly suppressed cell proliferation and promoted apoptosis in several cancer cell lines, as well as exhibited anti-cancer activities in different *in vivo* studies [19,20]. Cárdeno *et al.* explained that the anti-cancer effect of OLR was achieved by activating the p53 pathway and modifying the HIF-1 response to hypoxia. Additionally, OLR inhibits the development of HT-29 cells and causes apoptosis [21]. OLR has also been linked to chemopreventive activities in c57bl/6 mice with colitis-associated CRC [22]. These outcomes are connected to OLR's capacity to control gene expression and the activity of several signaling proteins implicated in apoptosis and proliferation [23].

Moreover, OLR showed a time and concentration-related cytotoxic impact against MCF-7 breast cancer cell lines via antiangiogenic and apoptotic effects through an increased PARP level with significant decrease in VEGF level [24]. Additionally, OLR induced apoptosis via abrogating NF-κB activation cascade in estrogen receptor–negative breast cancer cells [25]. Moreover, Scicchitano *et al.* confirmed that high concentrations of OLR showed anti-proliferative and pro-apoptotic activity on HEY and MCF-7 cells [26].

## Colorectal cancer

**Figure 1.**
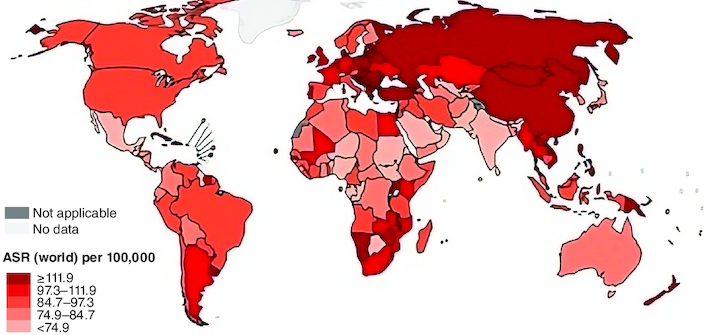
Estimated colorectum age-mortality worldwide rates in 2020. Reproduced from http://gobocan.iarc.fr.

Colorectal cancer (CRC) often referred to as colon and rectal tumors and called colon cancer, in which colon or rectum cells grow abnormally. Colon is the large intestine while the rectum is the passage that attaches the colon to the anus [27]. CRC is the third prevalence cancer worldwide, and the second cause of cancer death, comprising 11% of all cancer diagnoses (Figure 1) [28]. Surgery and chemotherapy can recovery more than two-thirds colon cancer patients and CRC patients appear to have a 5-year survival, but more than a third of these patients have a new neoplastic polyp, and 10% develop a second candid malignancy with a similar number of deaths for men and women [^29-31^].

### Prevalence

The 30% of patients with a family background of CRC cancer are at real risk of acquiring early CRC. The overall prevalence of hereditary CRC was 3–5%, and the prevalence was relatively high in patients older than 50 years of age. [32]. Prevalence of pathogenic germline mutations accounts for 16–25% of CRC patients [33]. In a prospectively identified study, testing of a polygenic panel (25 genes) specified germline mutations in 16% of patients, a third of patients with mutations did not meet genetic testing criteria based on the guidelines for the mutated gene; 25% of patients have hereditary colorectal cancer syndrome [34].

### Risk factors

In 1990, the highest proportions attributed to the risk factors for CRC were lifestyle and dietary intakes, low-milk diets and smoking, as low-milk diets outweighed risks from smoking, which were different from those reported in the analyzed data. In 2017, the three most important risk factors were low calcium diet, alcohol use, and no milk diet. Compared with 2019, the three highest CRC risk factors were low whole grains (6.1–20.7%), low milk (9.9–20.7%) diet and smoking (8.6–17.5%). The related risk ratios for CRC increased due to a higher fasting plasma glucose and higher body mass index, while the proportions attributed to smoking, alcohol use and a diet rich in processed meat decreased and the ratio of risk factors contributing to CRC mortality differed by country and age. In 2019, the proportion attributed to higher fasting plasma glucose and lower levels of physical activity increased CRC, but the ratio of CRC decreased with low milk and calcium diets [35].

### Management & treatment options

Early detection of CRC is critical to survival. Early-stage CRC have a 95% 5-year survival rate. There are several active areas in the management of CRC patients such as lifestyle interventions, chemotherapeutic drugs and specific treatments such as radiotherapy and surgery [36]. In several cases, surgery is expected to completely eradicate the tumor, but approximately a 25% of CRC cases are diagnosed at the advanced stage. so curative surgical control alone is often difficult, leading to deaths [37]. To shrink or stabilize a tumor, radiation therapy or chemotherapy may be used before or after surgery. Current chemotherapy includes treatment with a single agent (mainly fluorouracil [5-FU]) and multiagent regimens, including oxaliplatin (OX), irinotecan (IRI) and capecitabine (CAP). The dual therapy of 5-FU and OX, 5-FU and IRI, CAP and OX or CAP and OX are still the dominant first-line treatment approaches. Individual therapy is recommended for patients with poor functioning or a with low risk of deterioration. Additive agents options improving the therapy effectiveness, altering the adverse effects [38]. Nevertheless, there are still several irreversible obstacles, as systemic poisoning, unsatisfactory rejoinder rates, fluctuating instinctive and acquired resistance and low tumor selectivity. As a result, large efforts and money have been invested in creating novel approaches to improve or even replace standard CRC chemotherapy. Several new approaches for CRC treatment and managements involve immune and gene therapy [37].

#### Targeted therapy

Targeted therapies slow the growth and spread of tumor cells and limit damage to healthy cells. This therapy targets specific cancer genes, enzymes, or receptors that are involved in the growth and survival of cancer cells [39].

#### Gene therapy

Gene therapy targeting defective genes like TP53 and KRAS in colorectal cancers possibly acts as an alternate therapy for the disease in side to chemotherapy [40].

#### Immunotherapy

Medicines that promote the immune system to destroy cancer cells. Immunotherapy, also called checkpoint inhibitors, may be suitable when CRC cells show particular genetic modifications and characteristics [41]. Table 1 represent the FDA-accepted immunotherapy drugs for CRC [37,42].

**Table 1.** Colorectal cancer US FDA approved immunotherapy.

Immunotherapy	Receptor	Target
Cetuximab	EGFR	Transmembrane protein
Bevacizumab or panitumumab	EGFR, VEGF/VEGFR	Transmembrane/signaling protein
Ziv-aflibercept or regorafenib or ramucirumab	VEGF/VEGFR	Signaling protein
Pembrolizumab or nivolumab or ipilimumab	Block PD-1	Programmed cell death protein 1

#### Adoptive T-cell therapy

Adoptive T-cell therapy promotes immunity against tumors and increases vaccine efficacy. Recently, researchers have focused on saturating effector T cells with antigenic targets of interest, such as: B. CAR T cells. T cells showed extraordinary therapeutic potential [43].

#### Cytokine therapy

Cytokines are vital parts of tumor immunity, particularly in CRC, where the inflammatory process and immune stimuli lead to tumor progression [44]. Tumor necrosis factor and IL-6 are critical for CRC because it induce oncogenic factors, nuclear factor B and inducer of transcription 3 (STAT3) in GIT cells, which promotes proliferation and resist apoptosis [45]. Table 2 represent recent studies targeting CRC cytokine signaling pathways [46].

**Table 2.** Represent recent studies targeting colorectal cancer cytokine signaling pathways.

Cytokine	Potential therapy	Intervention
TNF	INCAGN01876 Vactosertib	Stimulation Inhibition
TGF	NIS7933, AP 12009 and anakinra	Inhibition
IL-1	CAN04	
IL-7	NT-17, GM-CSF and leukine	Stimulation
GM-CSF	Sargramostim	
IFN-γ	JX-594, GVAX and IFN-γ	

#### Natural products

**Figure 2.**
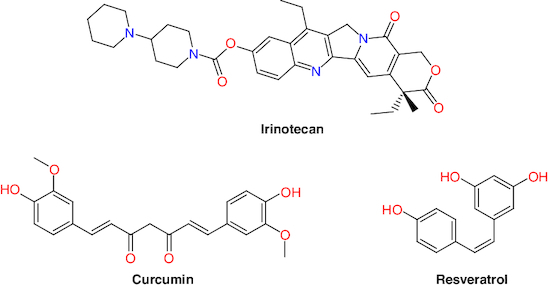
Structures of some natural products drugs used in colorectal cancer.

Nature provides first-line treatment sources and promising compounds for the treatment of many severe disorders [47]. Several plant compounds are in various stages of clinical trials for CRC treatment, including andrographolide, berberine, curcumin, epigallocatechin gallate, metformin, methotrexate, resveratrol, silymarin, SN-38, irinotecan and topotecan (Figure 2) [48,49]. These natural products have the potential to provide anti-CRC agents by interfering with the progress pathways of the secondary malignant growths, invasion, apoptosis and angiogenesis [50].

Interestingly, Mediterranean diet (MD) is associated with a low incidence of CRC. Storniolo *et al.* investigated the action of Sofrito components (an MD food preparation depends on extra virgin olive oil) on reactive oxygen species and eicosanoid production as well as the cell growth/cell cycle in adenocarcinoma cell cultures. They reported that that hydroxytyrosol (HOL), naringenin, naringenin glucuronide and lycopene and β-carotene modulate these events in Caco-2 cell cultures [51].

## Oleuropein against CRC

### Olive oil contents

Olive oil (OO; Olea europaea; Oleaceae) is a main component of the Mediterranean diet. Fatty acids like oleic and linoleic acid, secoiridoids like OLR and oleocanthal, simple phenols like tyrosol and hydroxytyrosol (HOL), lignans like pinoresinol, flavonoids like apigenin and hydrocarbons are all part of the complex chemical makeup. The extraction method for olive oil affects the chemical composition of OO. High pressure is applied to crushed olives to extract the oil without damaging the fruit pulp. Alternatively, you can use extrusion, post-pressing or re-pressing with hot water. Made in different ways, OO is more saturated in color, has a more pronounced odor and a larger amount of free fatty acids. Olives that are used to produce virgin olive oils (VOOs) have not been processed in any way that would alter the oil's original flavor or consistency. Cold-pressed, non-fermented olives are the source of all extra-virgin olive oils (EVOOs). The phenol content is extremely high, while the free fatty acid content is less than 1% [52].

There is a large quantity of wastewater, known as olive mill wastewater (OMWW), produced during OO extraction process as a byproduct of separating the oil from the paste. OMWW is a pollutant, yet its extract is high in polyphenols, therefore it's not all bad. The phenolic proportion in OO varies from 50 to 800 mg/kg based on several factors, including but not limited to: the season, the cultivar used, the degree of ripeness of the drupes during harvest, and the method of production. Virgin olive oil or ideal olive oil numerous studies have shown that the OO compounds, notably the phenols, have beneficial effects on free radicals, inflammation, the gut microbiome and cancer formation [1].

Many chemicals found in OO diets that showed anti-tumor activity *in vitro* cannot be consumed at the same concentration *in vivo* with an OO or supplement diet. Due to poor absorption and rapid metabolism, polyphenols had a limited bioavailability and were excreted in the urine. Despite extensive study over the past few years, the bioavailability of most OO polyphenols remains a mystery [53].

### Chemistry of oleuropein

The olive (Olea europaea) tree's leaf and fruit, as well as the extra virgin olive oil, are rich sources of oleuropein (OLR). OLR is nonflavonoid biophenol. Glucosylated elenolic acid and 3,4-dihydroxyphenylethanol form the heterosidic ester known as OAOL. The chemical is abundant in the fruits, which accounts for their bitter flavor [54].

**Figure 3.**
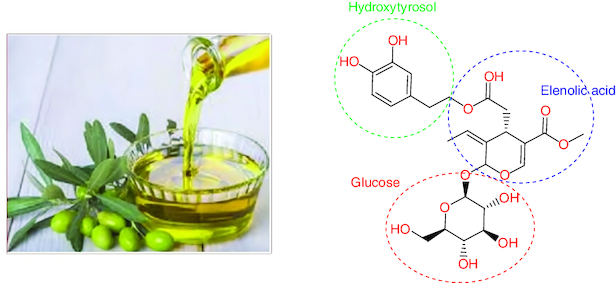
The chemical structure of oleuropein.

OLR has a wide variety of potential architectural applications (Figure 3). Comparing the structures of OAOL and decarboxymethyl oleuropein aglycone (DOA), for instance, reveals that they share a dihydroxylated aromatic moiety [55]. The methoxycarbonyl group found on C-5 of OA's dihydropyrane ring is what sets it apart from OLR. There are many different isomers of it since the iridoid moiety can take on a variety of configurations. However, the monoaldehydic type of olive oil is always the most common. They found that there were three main diastereomers, and they were (5S), (8R) and (9S) (5S, 8R and 9R) [56].

### Receptors involved in OLR action against CRC

Multiple investigations using several cell lines have demonstrated that OLR inhibits cell proliferation. Findings suggested that OLR inhibited NF-κB and its downstream targets cyclin D1 and cyclooxygenase-2 (COX2). In the graphic below, NF-κB activation cascade components Akt and IB are shown to have their expression reduced by OLR, which is considered to be responsible for this effect [57].

The COX2 pathway is connected to CRC because it promotes proliferation and angiogenesis via increased prostaglandin production. OLR was demonstrated to downregulate COX2. The suppression of cAMP response element-binding protein (CREB) is linked to this effect [58].

B-cell lymphoma 2 (Bcl2); NF-κB; the wnt/-catenin pathway; and peroxisome proliferator-activated receptor (PPAR) were discovered also linked to CRC. OLR, was reported to be responsible for upregulating PPAR gene expression in HT-29 colon cancer cells *in vitro* [59,60].

**Figure 4.**
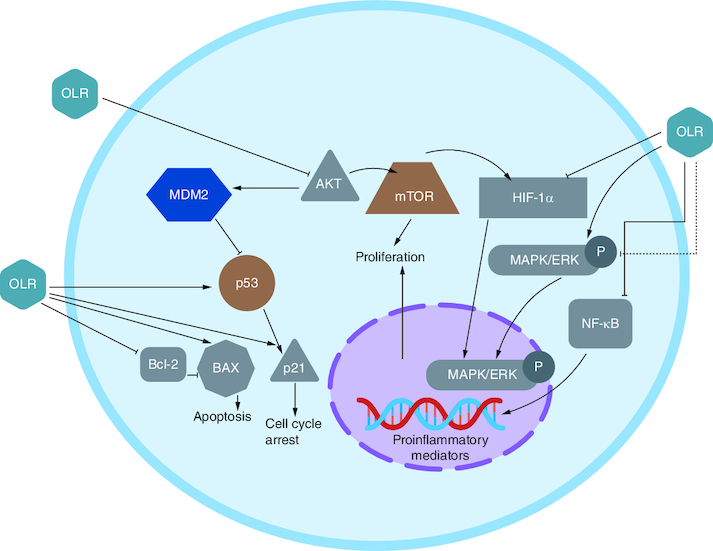
Receptors involved in oleuropein action against colorectal cancer.

OLR inhibited cells proliferation via blocking the NF-κB pathway. It has also been claimed that OLR did not affect IB in HT 29 colon cancer when administered at 400 and 800 M. Phosphorylation of inhibitor proteins, such as IB, is a common mechanism for activating NF-κB. On the other hand, this is not necessary for those following the alternative route (Figure 4) [57]. OLR caused apoptosis in colon tumors at 800 μM via raised the ratio of Bcl-2-associated X protein (Bax) and Bcl2, favoring the apoptotic pathway [61]. Additionally, OLR prevented CRC progression through upregulation of the gene coding for cannabinoid receptors (CB) [62].

### *In vitro* studies against colorectal cancer

Treatment of CRC SW620 cells with 10–100 lM OLR for 72 h considerably inhibited their proliferation compared with HT29 cells. Moreover, OLR induced apoptosis in both subtypes of colon cancer cells, which was linked with a block in the cell cycle (S phase). Similarly, the colorectal cell lines HT29 and SW480 were inhibited in their development and showed enhanced apoptosis after being treated with OLR (10–100 lM for 72 h). The G2M cell-cycle arrest was connected to this phenomenon. OLR (200–800 lM for 24, 48 and 72 h) dramatically inhibited the growth of HT29 human colon adeno-carcinoma grade II cells, indicative of the cell cycle halting (S phase). Additionally to inducing apoptosis, OLR upregulated p53 expression and downregulated HIF-1α protein expression [63].

In another study, HCT116 and RAW cells were exposed to 50 M OLR in full media for either 24 or 72 h. This concentration was determined to be safe after being verified on colon and macrophage cell lines in preliminary investigations. The NO release from RAW264 was analyzed to realize the role of OLR in the pro-inflammatory activity of macrophages. Seven cells were either subjected to LPS for 24 h after being treated with 50 M OLR (acute exposure) or were pretreated with 50 M OLR for 72 h (non-acute exposure). Resting RAW264.7 cells' NO creation was unaffected by either acute or chronic OLR administration. Short-term activation of RAW264.7 cells with OLR resulted in a 50% reduction in NO production relative to control cells. Long-term exposure to OLR reduced NO production in LPS-activated macrophages compared with their NO production when first activated by LPS. Similarly, both short- and long-term exposure to ORL significantly altered iNOS expression. Finally, OLR significantly altered COX-2 expression after only a brief period of exposure but had no effect after prolonged contact. It's worth noting that both iNOS and COX-2 expression levels were determined using the same tubulin reference and set of samples. Inhibiting iNOS and COX-2 may both contribute to the regression of a tumor lesion. In fact, high levels of iNOS can lead to the production of reactive mutagenic agents, which can harm DNA or make it more difficult to repair it. Tumor growth can be sustained through COX-2 stimulation [64].

One of the OLR metabolites is HOL (Figure 3). Bernini *et al.* investigated the effect of 6% HOL lipophilic derivatives on the Human colon cancer cell line HCT8-β8 modified to overexpress estrogen receptor β (ERβ). The result showed that all HOL fractions exhibited an antiproliferative effect and hydroxytyrosyl oleate showed the highest activity [65]. Moreover, Terzuoli *et al.* explored the role of HOL in the regulation of EGFR expression in human colorectal adenocarcinoma cells HT-29, CaCo-2 and WiDr, and in HT-29 xenografts. They found that HOL downregulate EGFR that associated with reduced cell proliferation that may be crucial for colon tumor management and treatment [66,67].

Llor *et al.* verified that OLR and HOL downregulated the expression of BCL-2 and COX-2 proteins that have a substantial role in CRC [68]. Gill *et al.*, verified that HOL and OLR suppressed the initiation, advancement and metastasis stages of colorectal carcinogenesis. DNA damage was lowered in HT29 human colon cells by elevating cellular capacity to fight against damage. An important increase was also detected in the barrier function of CaCo-2 cells. They also decreased the invasiveness of HT115 CRC cells metastasis [15,69]. HOL also activated the phosphorylates FOXO3a, phosphoinositide 3-kinase/Akt pathway, induced the cell death and mitochondrial dysfunction and then downregulated FOXO3a's target genes in human CRC cells (DLD1) but not on normal colon cells (1807) by producing ROS in CRC cells [70].

In another study when HT-29 colon cancer cells treated with increasing OLR concentrations, OLR suppresses the proliferation of CRC cells by preventing DNA generation and protein expression of CYP1A1, GSTM1 and NQO1 enzymes [71].

Corona *et al.* examined the anti-proliferative properties of an olive oil polyphenolic extract 50 μg/ml) including OLR on human CRC cells. The result showed a robust inhibitory effect on cancer cell proliferation related to the induction of a G2/M phase cell cycle block. This block was refereed by the ability of 50 μg/ml polyphenol extract to employ quick inhibition of 38.7% of p38 and 28.6% of CREB [72].

Furthermore, the effect of OLR on cell proliferation was evaluated on colorectal adenocarcinoma-supraclavicular region metastasis (LoVo). LoVo cells were incubated with increasing concentrations of OLR (0.005–0.025%). After 5 days, the result showed that 0.1% OLR completely blocked the invasion of tumor cells through a thick, undiluted Matrigel layer to the other side of the filter membrane and OLR-treated spheroids were damaged and were unable to move through the matrix [73]. OLR also had a clear suppressive effect on tumor sphere development capacity and induced apoptosis in a concentration-dependent manner. In addition, combination treatment with 5-FU and OLR revealed a synergistic effect on cell viability in DLD-1 cells and 5-FU-resistant cells [74].

Nevertheless, oleic acid consumption has been reported to act as a potent mitogen in Caco-2 cells and induce CRC. HOL, OLR and pinoresinol reversed DNA synthesis and Caco-2 cell proliferation induced by oleic acid [75]. OLR effects can be related with HOL release because of OLR hydrolysis by Caco-2 cells (up to 25%). Furthermore, HOL modulates the arachidonic acid cascade, and this event can be associated with its antimitogenic action [76].

### *In vivo* studies against colorectal cancer

In A/J mice with azoxymethane (AOM)-induced leukocyte damage of DNA, researchers found that supplementing the mice's basal diet with OLR (125 mg/kg, 7 and 17 weeks) significantly reduced the severity of crypt dysplasia and the number of tumors in the colon's middle third [77].

Together, dextran sulphate sodium (DSS) and azoxymethane (AOM) were used to promote inflammation and cancer in mice (C57BL/6). Increases in inflammatory markers in colon tissue were detected by testing for IFN-c IL-6, IL-17A, TNF-α and COX-2. When these animals were given OLR (50 or 100 mg/kg b.w.), inflammation markers decreased and the amount of colon tumors decreased (by 64 and 16%, respectively). In addition to reducing tumor size and number, OLR also inhibited the growth of cancer cells. There was a correlation between the effects of OLR and increased Bax expression and decreased Ki-67 expression. Additionally, it inhibits phosphorylation of NF-κB, Akt, Wnt/-catenin, and STAT-3. [78].

Moreover, Giner *et al.* investigated the effect of OLR on dextran sulfate sodium (DSS)-induced experimental colitis in mice. OLR reduced the extent and severity of acute colitis while minimizing neutrophil infiltration; formation of NO, IL-1β, IL-6, and TNF-α; expression of iNOS, COX-2 and MMP-9; and the translocation of the NF-κB p65 sections to the nucleus in colon tissue. The results showed that OLR effect on DSS-induced colitis is associated with a decrease in the formation of interleukins and expression of proteins, primarily through reduction of NF-κB activation [79].

Furthermore, OLR prevented the incidence of CRC in the azoxymethane (AOM)/DSS model in C57BL/6 mice. OLR blocked the growth and diversity of colonic tumors (84%), lowered COX-2 (70%) expression, and decreased nuclear p65 NF-κB subunit (49%). Moreover, OLR regulated apoptotic proteins as Bax (30%), lowered the translocation of β-catenin (49%) to the nucleus and the activation of pathways associated on tumor growth: AKT/PI3K (40%) and STAT-3 phosphorylation (35%) [80].

Another study was performed to reduce the risk of CRC due to AOM/DSS, OLR alleviated symptoms and reduced the disease activity index score. Also, it stifled the multiplication of tumors in the colon. Intestinal IFN-, IL-6, TNF-, as well as IL-17A levels were all reduced after OLR administration, as was the expression of cyclooxygenase-2, Bax, and proliferating cell nuclear antigen protein. An increasing DAI score was associated with this variable, suggesting that clinical symptoms including diarrhea and rectal bleeding. Both 50 and 100 mg/kg OLR were effective in reducing body weight loss and diarrhea/rectal bleeding after the initial DSS cycle. This shielding persisted during the experiment, and there was a discernible trend toward weight stability and decreased diarrhea and feces-borne blood in the DAI score. Mice were slaughtered on day 63. Compared with mice administered OLR, animals given DSS or AOM/DSS had a shorter colon and a higher W/L ratio. The clinical indications worsened and 40% of the mice died after the first cycle of 5-ASA treatment. The mice that made it through the second cycle, however, fared far better, both in terms of weight increase and DAI score improvement. Mice in groups C, A,and O100 of the AOM/DSS study lost no weight and displayed no clinical signs, earning a DAI score of 0 [78].

Another study was performed *in vitro* for Olive oil by Valaei *et al.* Sixty male Wistar rats, weighing between 200 g, were examined in the experiment. Using the GC-MS technique, we were able to identify the individual components of a MOS, or a mixture of olive and sesame oil extracts. Thereafter, various markers associated with colon cancer remission and progression were sought out using homogenates of colon tissue. Standard pathological abnormalities in colon tissue were used to compare the groups, including necrosis, mitotic index and inflammatory cell infiltration level. Findings revealed no distinction in necrosis, inflammatory cell, or mitotic activity levels between the control, MOS and olive oil groups (p more than 0.05). While the first three groups' indices were similar, the DMH group's indices were vastly different (p more than 0.05). After scoring and staging, it was determined that the DMH + MOS-exposed group had much less necrosis, inflammatory cell infiltration and mitosis than the DMH group [81].

Sepporta *et al.* explored the ability of OLR to stop the azoxymethane (AOM)-induced colon cancer upset and DNA disruption in mice. An OLR-enriched diet prevented the AOM-induced preneoplastic lesions in different colon segments, decreasing the severity of crypt dysplasia and DNA damage in peripheral leukocytes. OLR can prevent CRC and DNA damage in mice treated with the carcinogen AOM [77].

Moreover, Schwingshackl *et al.* discovered that the most stringent adherence to a Mediterranean diet was linked with the lowest risks of dying from and getting various types of cancer. However, research on olive oil's efficacy as a standalone ingredient revealed no significant reduction in cancer risk. However, the studies pooled together for this meta-analysis were sparse on specifics, such as participants' adherence to the Mediterranean diet or the relationship between dietary styles and bioactives. This meant that such intricate connections could not be explored in the current meta-analysis. We discovered that coastal countries benefited from the improvements we found in our meta-analysis, even though the proportions of different food groups and how they affect health are likely to be different from country to country [82].

## Toxicity aspects

According to bioavailability studies, OLR is absorbed to a greater extent than 55–66 mol% in humans, and at least 5% is eliminated in the urine. moreover, in less than 2 h, OLR and its metabolites are identified at their highest levels in serum and urine. OLR is therefore thought to be highly safe [83]. High concentrations of OLR administered *in vivo* were demonstrated to be safe, and studies on rodents showed that it had an excellent safety profile [84].

OLR's toxicity as well as that of its two primary metabolites, HOL and elenolic acid (Figure 3), were all proven to be safe in a variety of animal kinds. OLR's acute toxicity trials did not show any fatality or negative effects in mice, even at concentration of 1000 mg/kg. Additionally, normal embryo development was observed when injected OLR into fertilized chicken eggs [73]. Moreover OLR may work synergistically with current forms of chemotherapy to diminish the dosages that frequently cause toxicity and significant side effects [23].

To prevent antagonistic or additive drug interactions and side effects, patients should see their doctor before using OLR. OLR may worsen low blood pressure status in patients who already have it. When administered via intravenous or intraperitoneal injections, OLR drastically lowers both systolic and diastolic blood pressure in animal models [20].

## Conclusion

OLR was proven to prevents cell proliferation by interacting with the AKT/NF-κB pathway and COX2, PPAR, MMP and CB receptors. OLR decreases cell viability and causes cell cycle arrest along with antioxidant characteristics. OLR has a good synergistic effect when merged with anticancer agents. OLR is a good candidate that could be used as a supplement along with anticancer therapy as a preventive therapy.

## Future perspective

Due to the extraordinary therapeutic and preventive effect of OLR, we need to further exploit this key Mediterranean dietary component to promote human health.
